# Modulating the thermal and structural stability of gallenene *via* variation of atomistic thickness†

**DOI:** 10.1039/d0na00737d

**Published:** 2020-12-23

**Authors:** Stephanie Lambie, Krista G. Steenbergen, Nicola Gaston

**Affiliations:** Department of Physics, MacDiarmid Institute for Advanced Materials and Nanotechnology, University of Auckland Private Bag 92019 Auckland New Zealand n.gaston@auckland.ac.nz; MacDiarmid Institute for Advanced Materials and Nanotechnology, School of Chemical and Physical Sciences, Victoria University of Wellington P.O. Box 600 Wellington 6140 New Zealand

## Abstract

Using *ab initio* molecular dynamics, we show that a recently discovered form of 2D Ga—gallenene—exhibits highly variable thickness dependent properties. Here, 2D Ga of four, five and six atomic layers thick are found to be thermally stable to 457 K, 350 K and 433 K, respectively; all well above that of bulk Ga. Analysis of the liquid structure of 2D Ga shows a thickness dependent ordering both parallel and perpendicular to the Ga/vacuum interface. Furthermore, ground state optimisations of 2D Ga to 12 atomic layers thick shows a return to a bulk-like bonding structure at 10 atoms thick, therefore we anticipate that up to this thickness 2D Ga structures will each exhibit novel properties as discrete 2D materials. Gallenene has exciting potential applications in plasmonics, sensors and electrical contacts however, for the potential of 2D Ga to be fully realised an in depth understanding of its thickness dependent properties is required.

## Abstract 2

Using *ab initio* molecular dynamics, we show that a recently discovered form of 2D Ga—gallenene—exhibits highly variable thickness dependent properties.

## Graphical abstract

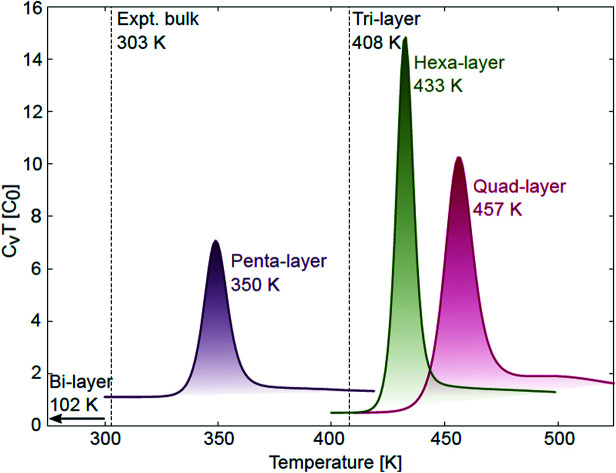

## Introduction

Gallium (Ga) has an array of applications in the current technological age, being used as a component in light emitting diodes,^1^ reversible light-induced switching,^2^ phase-change non-linear systems,^3^ active plasmonics,^4^ chemical sensing,^5^ molecular sensing,^6^ and for drug delivery.^7^ Ga is considered a technology-critical element.^8^

Despite Ga's use in a wide variety of everyday technologies, Ga is an unusual element because it is highly polymorphic. α-Ga is the standard phase and is stable at atmospheric pressure and room temperature, however, a range of other phases (β, γ, δ) are accessible at a variety of temperatures, while Ga(i), Ga(ii) and Ga(iii) exist under different pressures.^9^ Furthermore, Ga has a hugely complex phase diagram with one of the largest liquid temperature ranges of all the elements.^10^

Bulk α-Ga adopts an orthorhombic unit cell, consisting of 8 atoms.^11^ Each Ga has one nearest neighbour at approximately 2.44 Å forming covalently bound dimers, while the remaining 6 nearest neighbours are metallically bound in a strongly buckled plane perpendicular to the average alignment of the dimers.^12–14^ Bulk α-Ga has a low melting temperature (*T*_melt_) of 303 K, putting Ga in a select group of low-melting temperature metals (where “low-temperature” is defined as *T*_melt_ < 303 K) which includes only four elements; Hg, Cs, Fr and Ga. The low *T*_melt_ of Hg and Cs is attributed to a low cohesive energy.^15,16^ For Hg, this correlation was strengthened by a first-principles investigation of relativistic effects.^17^ However, Ga has a cohesive energy comparable to that of Al (*T*_melt_: 934 K) and In (*T*_melt_: 430 K).^18^ Typically, bulk α-Ga's low *T*_melt_ is attributed to the dimeric Ga_2_ bonding structure.^19^

An exciting new avenue for Ga to be used in electronic applications has recently developed, with experimentalists successfully synthesising two-dimensional (2D) Ga through solid-melt exfoliation.^20^ 2D Ga, in keeping with bulk Ga, is unique among 2D materials. Typically, 2D materials are held together by strong covalent bonds in-plane but only weak van der Waals interactions between the layers as in the case, for example, of graphene.^21,22^ In contrast to this, 2D Ga is formed by cutting through covalently bound dimers, while maintaining metallic behaviour in plane.^20,23,24^ Furthermore, in the bi-layer, (two atomic layers thick) this metallic character has been shown to be robust to significant lattice strain and therefore is expected to persist even when supported by a wide range of substrate materials.^23^ Due to 2D Ga having dangling covalent bonds, this material is able to bond covalently to a substrate material.^20,25,26^ However, an important property of 2D Ga that remains unexplored before it can be used effectively in electronic applications is a robust understanding of the material's thermal stability.

Ga nanoclusters are known to exhibit hugely variable thermal stability at the nanoscale.^19,27–30^ At the nanoscale, the melting temperature (*T*_melt_) is dictated by:1
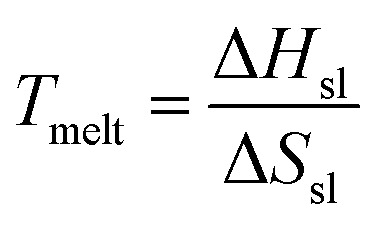
where Δ*H*_sl_ is the change in the enthalpy and Δ*S*_sl_ the change in entropy between the solid and liquid state. Liquid Ga nanoclusters have been shown to exhibit ordering which results in a reduction in entropy of the liquid state, which leads to a lower Δ*S*_sl_ and correspondingly increases the *T*_melt_. Recently, the thermal stability of the bi-layer and tri-layer (three atomic layers) 2D Ga was investigated computationally by our group. It was found that the tri-layer structure is thermodynamically stable to 306 K higher than the bi-layer structure.^24^ Furthermore, studies centering around the liquid phase of bulk Ga have shown liquid–liquid phase transitions,^31–35^ a tri-layered liquid structure at the free surface^36^ and structural ordering at high pressure and temperature.^37^

Ordering in 2D liquid phases has a long history. In the 1970's, Kosterlitz, Thouless, Halperin, Nelson and Young developed KTHNY theory which describes melting of 2D systems as the decoupling of pairs of topological defects and predicts an intermediate phase between solid and liquid called the “hexatic-phase”.^38–42^ In a solid 2D system, the crystal has long-range orientational order and quasi-long range translational ordering as a result of reduced dimensionality, the hexatic phase exhibits quasi-long range orientational order and short range translational order while in the liquid phase both translational and orientational order are short range.^43,44^ Although KTHNY theory and the existence of the hexatic-phase were originally developed for extended 2D systems, without consideration of free surfaces^45^ the hexatic phase has been positively identified in supercooled liquid metal surfaces.^46^

In this study, we extend the previous work by our group on the thermal stability of 2D Ga by using *ab initio* molecular dynamics (AIMD) simulations to determine the *T*_melt_ of 2D Ga as the thickness is increased from four (quad-layer), to five (penta-layer) and six (hexa-layer) atomic layers. Furthermore, we explore the relevance of the hexatic phase in the thermal stability of these systems.

## Methodology

All calculations use plane-wave density functional theory (DFT) within the Vienna *ab initio* Simulation Package (VASP)^47^ and the projector-augmented wave (PAW) method.^48^ For all calculations, a plane wave cut off of 350 eV was used. Methfessel–Paxton (order one) smearing with a width of 0.05 was used and all calculations were converged electronically to 1 × 10^−6^ eV. We use the Perdew–Burke–Ernzerhof for solids (PBEsol) exchange-correlation functional.^49^ For a variety of elements PBEsol has been shown to be effective at predicting melting temperatures in agreement with experiment.^50^ The thermal stability of bulk Ga has only been calculated using PW91 and was found to be 212 K;^29^ 91 K below the experimental bulk *T*_melt_. Interestingly, it has been shown for Ga_20_^+^, Ga_32_^+^, Ga_34_^+^ and Ga_35_^+^ nanoclusters that adding +90 K to the calculated PW91 melting temperatures provides agreement with experimentally yielded results (it should be noted, however, that there are two exceptions).^28^ Further, tri-layer calculations undertaken by our group found that the using PW91 the tri-layer melts 91 K lower than using PBEsol.^24^ Assuming that the melting temperature difference between PW91 and experiment of 90 K is consistent between nanoclusters and 2D Ga, we conclude that PBEsol provides results that are comparable to within 1 K of experiment.

### Optimisations

**Fig. 1:**
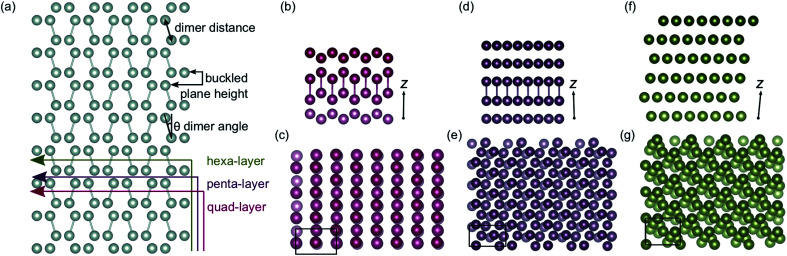
(a) Bulk α-Ga supercell with illustrations showing the cuts used to obtain the initial quad-layer (red line), penta-layer (purple line) and hexa-layer (green line) structures used to seed the MD simulations. The dimer distance, buckled plane height and dimer angle parameters are also defined. The optimised lowest energy Ga structures resulting from a phase change at temperature for (b) and (c), the quad-layer, (d) and (e), the penta-layer and (f) and (g), the hexa-layer. Perspectives along the *x*-axis, with the *z*-axis marked are shown in (b), (d) and (f) and perspectives along the *z*-axis are shown in (c), (e) and (g). The black boxes in (c), (e) and (g) show the unit cell. The cut off for the bonds was set to 2.7 Å.

The (010) termination of α-Ga was used to build structures that enabled the AIMD simulations to be seeded (see ESI† for more details). The initial α-Ga crystal was taken from the Crystallography Open Database with unit cell dimensions of 4.527 × 7.645 × 4.511 Å.^11^ Surfaces of four, five and six atomic layers were built containing eight, ten and twelve atoms per unit cell, respectively (Fig. 1a). Non-spin polarized optimisations of the structures were carried out using a Monkhorst–Pack *k*-point grid of 7 × 7 × 1 and *k*-point converged to within 0.1 kJ mol^−1^.

### Annealing

Supercells of each surface were created by repeating the optimised (010) α-Ga surfaces by 4 × 4 × 1, resulting in supercells of 128, 160 and 192 atoms for the quad-, penta- and hexa-layer systems, respectively. Single point *Γ*-centred *k*-points were used for all AIMD simulations. The annealing calculations were run in a *n*PT ensemble using the Parrinello–Rahman method^51,52^ with a Langevin thermostat. The *n*PT simulations were used only to equilibrate the structures at a range of finite temperatures and 0 kB pressure. The structures were annealed by incrementing the temperature by 80 K over 0.9 ps sequentially using a timestep of 3 fs until a melting transition was provisionally observed using root mean square displacement (RMSD) analysis. High temperature solids were then equilibrated for 9 ps before finite temperature melting simulations were seeded.

### Melting simulations

Finite temperature melting simulations were calculated within the *n*VT ensemble. Temperatures were selected to cover a minimum 100 K range at 20 K intervals with a timestep of 4 fs. For the quad-layer, temperatures ranged from 410–550 K, for the penta-layer, temperatures simulated were 300–420 K and for the hexa-layer, calculated temperatures ranged from 400–500 K. The AIMD simulations were run for a minimum of 100 ps per temperature. Specific heat curves were calculated using the multiple histogram method^53^ using the final 64 ps of each simulation to allow sufficient time for the structure to equilibrate and at 20 K increments to provide sufficient overlap of the finite temperature histograms.

## Results and discussion

### 2D polymorphism

As the quad-, penta- and hexa-layer systems were annealed, all systems changed phase to a lower energy structure (Fig. S1†). The systems changed phase at different temperatures (82 K, 124 K and 67 K, for quad-layer, penta-layer and hexa-layer, respectively) and changed to structures distinct from each other (Fig. 1b–g).

In order to quantify the structural change resulting from the phase-change, three parameters in bulk α-Ga are defined; dimer distance, dimer angle and buckled plane height (Fig. 1a). For bulk α-Ga, the dimer distance is 2.49 Å, dimer angle, relative to the *z* axis, is 17.3° and the buckled plane height is 1.44 Å (Table S2†). In the quad-layer structure, the dimers are maintained, although the dimer distance is extended to 2.68 Å while the dimer angle is significantly reduced to 1.0° (Fig. 1b and c). The buckled plane height varies from a maximum of 3.39 Å to a minimum of 1.86 Å (Table S2†) due to the corrugation in metallic surface layers and the offset of Ga_2_ dimers in the *z* direction. The radial distribution function (RDF) shows that the quad-layer is not geometrically similar to either the bulk α- or β-Ga (Fig. S2a†).

In the penta-layer structure, the phase change increases the dimer distance to 2.65 Å, reduces the dimer angle to 0.7° and increases the buckled plane height, which, in this case, is simply the plane height as the planes no longer buckled, to 2.41 Å (Fig. 1d and e, Table S2†). The RDF analysis does not show distinct similarity to either bulk α- or β-Ga (Fig. S2b†).

The optimisation of the hexa-layer phase-change structure results in the expansion of the system in the *z* dimension, such that no two Ga atoms are within 2.7 Å of one another. Thus, in the hexa-layer system, there are no Ga dimers analogous to those seen in bulk α-Ga (Fig. 1f and g) and the parameters used to define bulk α-Ga cannot be used to define the hexa-layer system. We note, however, that the interlayer distance stays reasonably constant between all of the layers at approximately 2.5 ± 0.1 Å. The RDF analysis shows that the hexa-layer system is not similar to bulk α- or β-Ga (Fig. S2c†).

We interpret the reduction in dimer angles and the extension of the interlayer distances from the bulk in all three of the systems to indicate that they are adopting a more traditional metallic-like close packed structure.

### Electronic structure

**Fig. 2:**
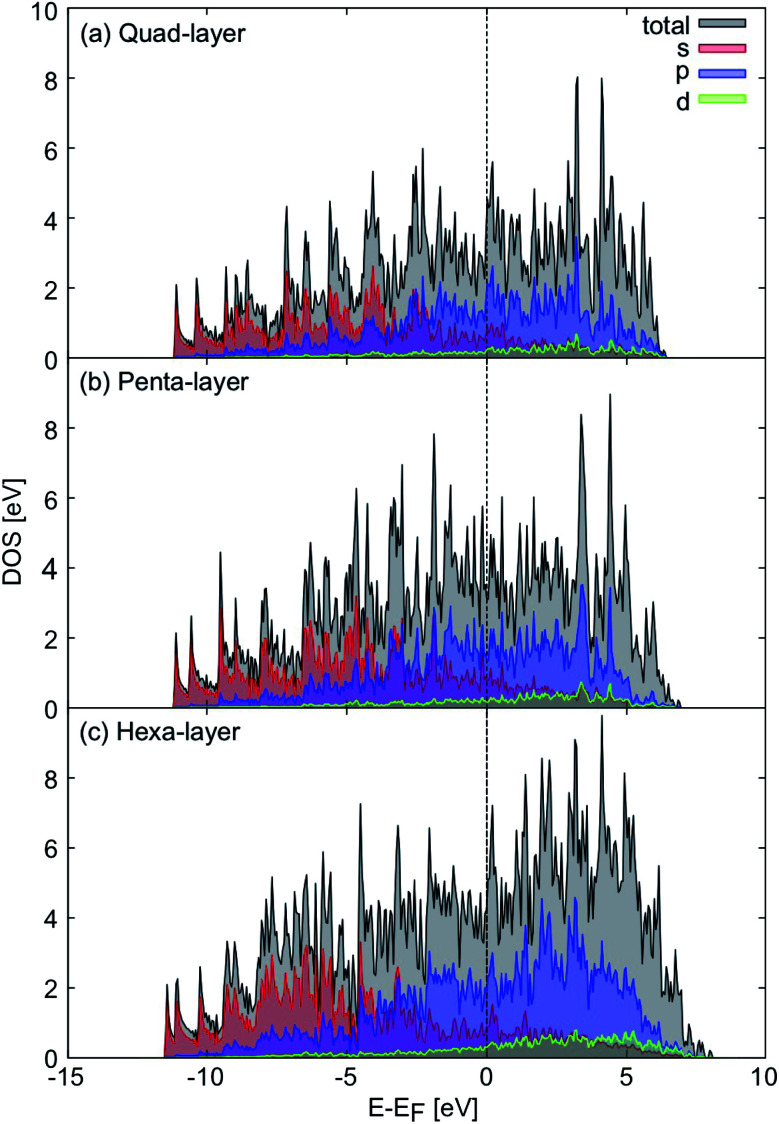
Projected density of states, relative to the Fermi level (black dotted line) for the (a) quad-layer, (b) penta-layer and (c) hexa-layer structures. Total density of states is shown in black, s orbitals are shown in red, p orbitals in blue and d orbitals in green.

Considering the electronic properties of the quad-layer system, the projected density of states (Fig. 2a), electron localisation function (ELF) analysis (Fig. S3b†), and band structure (Fig. S4a†) show that the lowest energy quad-layer structure is fully metallic as there is no band-gap present in the density of states which might indicate a different electronic behaviour of this system. Furthermore, there is no α-Ga-like pseudogap apparent in the density of states at the Fermi level.^54^

Evidence for fully metallic behaviour in the penta-layer structure is provided by the projected density of states (Fig. 2b). This analysis is reinforced by the ELF analysis which shows there is no covalent bonding in the penta-layer structure (Fig. S3c†) and by the band structure which shows metallic character (Fig. S4b†). As with the quad-layer system, the density of states shows no α-Ga-like pseudogap^54^ or band-gap, characteristic of an semi-conductor or insulator.

The density of states show that the hexa-layer system is also metallic (Fig. 2c) due to the lack of band-gap or pseudogap in the density of states, which is confirmed by the ELF analysis showing electron density localised on the atomic centres (Fig. S3d†) and the band structure (Fig. S4c†).

Based on the density of states, 2D Ga structures between two and six atomic layers are electronically indistinguishable (Fig. 2).^24^ Due to the fact that there is minimal variation in the electronic properties of 2D Ga systems up to six atomic layers thick, it could be advantageous to synthesise 2D Ga that is not ultra-thin. Using thicker forms of 2D Ga may enable the structure to be more robust for use in electronic applications.

The disappearance of covalency within 2D Ga structures, as evidenced by the ELF analyses (Fig. S3†) is not unprecedented. This phenomenon has also been observed in quasi-2D Ga nanoclusters (<100 atoms) where no α-like phase or covalent dimers are observed.^19^ Thus, the 2D Ga systems considered in this study have a closer structural similarity to quasi-2D nanoclusters than to bulk α-Ga. Interestingly, ELF analyses of optimised 2D slabs from a thickness of two to 12 atomic layers show that bulk-like structure and covalency reappears at a minimum thickness of 10 atomic layers (Table S3†).

**Fig. 3:**
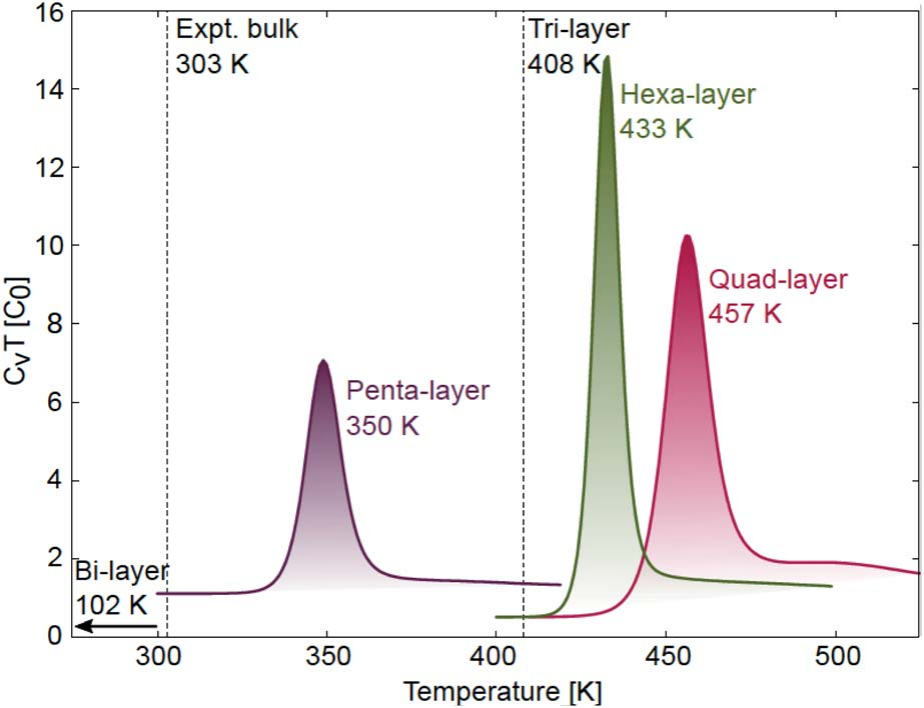
Specific heat curves for quad-layer (red), penta-layer (purple) and hexa-layer (green) 2D Ga with melting transitions observed at 457, 350 and 433 K, respectively. *T*_melt_ for the bi-layer (102 K) and tri-layer (408 K) are taken from ref. 24.

### Thermal stability

**Fig. 4:**
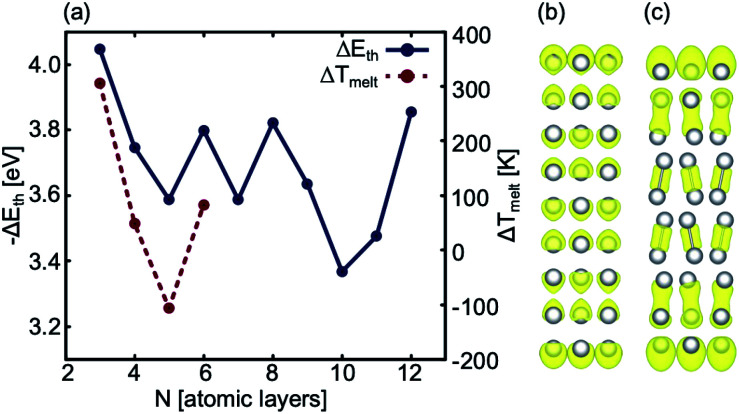
(a) Blue: The stability gained per atom as a result of adding a bulk atom to the system as the thickness of the slab is increased. Note that we use −Δ*E*_th_ to show this correlation. Red: Δ*T*_melt_ between 2D Ga systems with *N* and (*N* − 1) layers, (b) ELF analysis of a 9 atom thick 2D Ga structure, and (c) ELF analysis of a 10 atom thick 2D Ga.

The AIMD simulations find that the quad-layer system melts at 457 K, the penta-layer system at 350 K and the hexa-layer system at 433 K (Fig. 3). Therefore, 2D Ga from four to six atomic layers thick is thermally stable to well above the bulk Ga *T*_melt_. Furthermore, the variation in *T*_melt_ of the quad-, penta- and hexa-layer 2D Ga systems is not monotonic, nor does it converge toward the bulk *T*_melt_ with increasing thickness. Convergence towards the bulk *T*_melt_ is expected around a thickness of 10 atomic layers (∼2 nm), as this is where the ELF analysis shows the return of covalently bound Ga_2_ dimers characteristic of bulk α-Ga (Fig. 4b and c, Table S3†). We highlight that the return of bulk-like bonding at a thickness of ∼2 nm corresponds to the return of approximately bulk *T*_melt_ observed in Ga_94_ nanoclusters (∼2.4 nm in diameter), therefore loosely agreeing with our predictions here.^55^

Using ground state optimisations of 2D Ga systems, up to 12 atomic layers thick, we examine the stability gained from adding a bulk atom to the system as the thickness of the slab is increased, *E*_th_, using the following relation:2
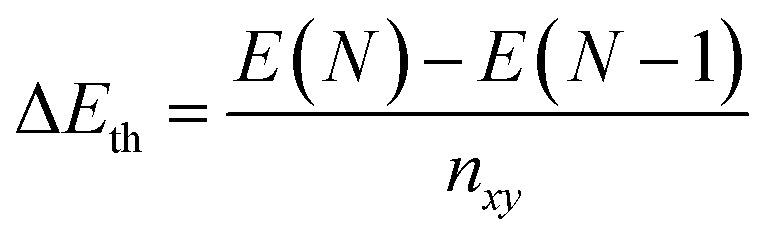
where *E*(*N*) is the total energy of an *N*-layered system, *E*(*N* − 1) is the total energy of an (*N* − 1) layered system and *n*_*xy*_ is the number of atoms in the *xy* plane. Above 10 atomic layers thick, we are cautious about making conclusions because at this thickness bulk-like bonding reappears in the systems (Fig. 4b and c). However, up to 6 atomic layers, if the energetic stability offered by adding a bulk atom to the system is large there is a correspondingly large change in the thermal stability of that system (Δ*T*_melt_ = *T*_melt_(*N*) − *T*_melt_(*N* − 1)) and *vice versa*. Therefore, we propose that up to 10 atomic layers thick, before the bonding structure reverts to being bulk-like, Δ*E*_th_ may provide a proxy for Δ*T*_melt_ in the 2D Ga structures. Furthermore, with the exception of increasing the thickness of the system from the bi-layer to the tri-layer, there is an increase in Δ*T*_melt_ offered by systems with an even number of layers over those with an odd number of layers and thus we determine that 2D Ga systems with an even number of layers have a higher thermal stability (Fig. 4a).

### Structural analysis of melting

**Fig. 5:**
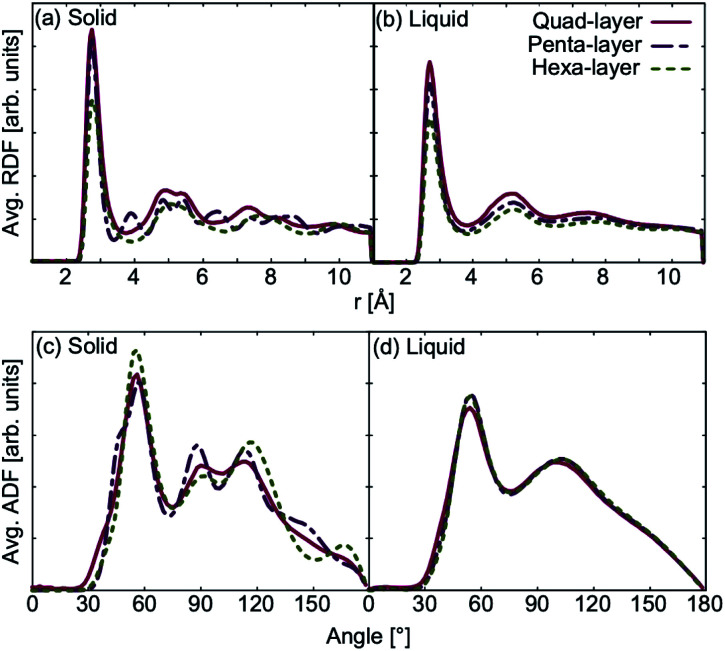
Average radial distribution function (RDF) for quad-layer, penta-layer and hexa-layer in the (a) solid phase and (b) liquid phase. Average angular distribution function (ADF) for quad-layer, penta-layer and hexa-layer in the (c) solid phase and (d) liquid phase. For the quad-layer (red), solid is defined as 410 K and liquid as 550 K; for penta-layer (purple), solid is 300 K and liquid is 420 K; for hexa-layer (green), solid is 400 K and liquid is 500 K.

The average RDF in the solid phase, shows that the quad-layer and hexa-layer system are very similar, while the penta-layer solid system exhibits more peaks (Fig. 5a). The angular distribution function (ADF) analysis in the solid state for all systems show peaks occurring at the same angles, varying only in their intensity (Fig. 5c). RDF and ADF analyses of the liquid state show analogous behaviour of all three systems (Fig. 5b and d).

Using the change in RDF and ADF between the high-temperature solid and liquid state as a rough proxy for the degree of structural reorganisation upon melting, the penta-layer system exhibits a greater structural change, corresponding to a greater change in entropy. From the *T*_melt_ relationship, eqn (1), it follows that a greater change in entropy upon melting leads to a lower melting temperature, agreeing with our results.

Average mean square displacement (MSD) analyses were used to examine the solid–liquid transitional structures. Transitional structures result from the finite temperature simulations closest to the calculated *T*_melt_ for each system; 450 K simulation for the quad-layer system (*T*_melt_ = 457 K), 340 K simulation for the penta-layer system (*T*_melt_ = 350 K) and 440 K simulation for the hexa-layer system (*T*_melt_ = 433 K). *x*- and *y*-dimensions are labelled as “in-plane” (repeated infinitely) while the *z*-dimension is finite and diffusion in this direction is termed “inter-planar.”

**Fig. 6:**
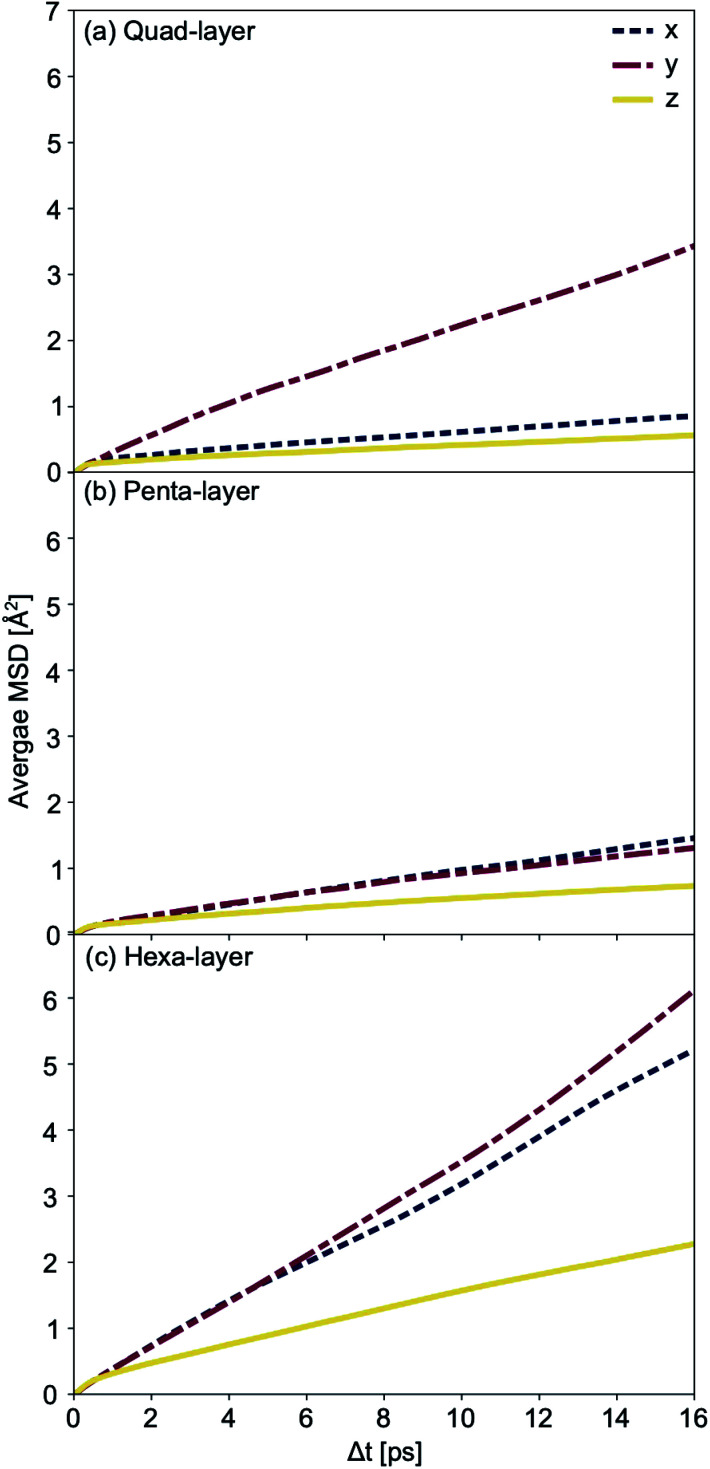
Average mean square displacement (MSD) for (a) quad-layer at an average temperature of 450 K, (b) penta-layer at an average temperature of 340 K and (c) hexa-layer at an average temperature of 440 K. MSD_*x*_ is shown in blue, MSD_*y*_ in red and MSD_*z*_ in yellow.

For the quad-layer system, the MSD_*y*_ is highest while MSD_*x*_ and MSD_*z*_ are considerably lower (Fig. 6a). Thus, it follows that atomic diffusion in the *y*-dimension is higher than in the *x*- and *z*-dimensions.

The penta-layer MSD analysis shows that MSD_*x*_ is roughly equal to MSD_*y*_, and both are considerably higher than MSD_*z*_ (Fig. 6b). Therefore, in-plane diffusion is roughly uniform, while inter-planar diffusion is limited.

The MSD analysis shows that, for the hexa-layer system, MSD_*y*_ is only slightly higher than MSD_*x*_ (Fig. 6c). MSD_*z*_ is low, in keeping with the quad- and penta-layer systems (Fig. 6c). Similar to the penta-layer system, MSD analysis of the hexa-layer system shows that in-plane diffusion is higher than inter-planar diffusion.

### Perpendicular surface ordering

**Fig. 7:**
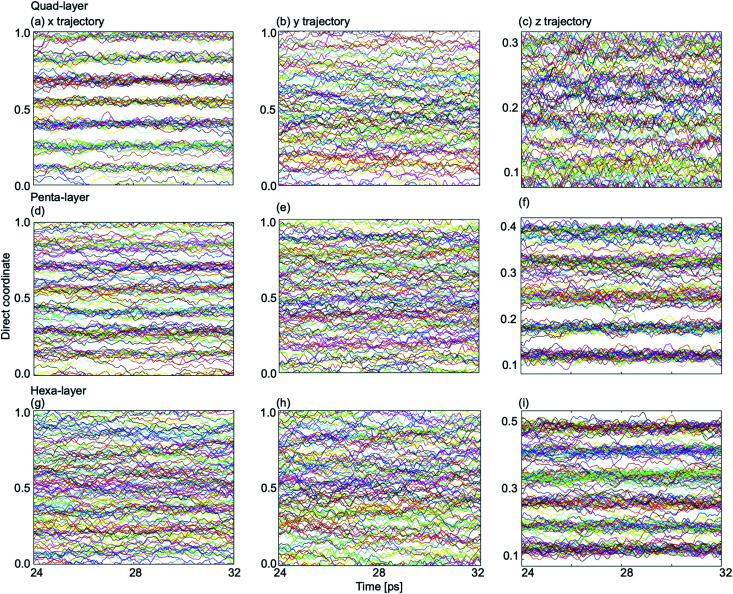
Single coordinate atomic trajectories in the *x*-dimension (left column), *y*-dimension (center column) and *z*-dimension (right column) for (a)–(c), the quad-layer system at 440 K; (d)–(f) the penta-layer system at 340 K; and (g)–(i) the hexa-layer system at 440 K. Note that the direct coordinates for the *z*-dimension are different due to vacuum requirements of modelling a 2D surface.

In order to explain the non-uniformity of in-plane atomic diffusion of the quad-layer system resulting from the MSD analyses, so-called “single-coordinate atomic trajectories” were plotted for all three systems. The single coordinate atomic trajectories show the *x*, *y*, and *z* positional coordinates of all of the atoms in the system over simulated time to provide structural information about the movement of the atoms in each dimension (Fig. 7 and S5–S7†).

In the quad-layer system, clear layering is seen in the *x*-dimension (Fig. 7a). We interpret this to mean that atomic diffusion in the *x*-dimension is limited, thereby explaining the low MSD_*x*_ (Fig. 6a). We attribute the lack of definition in the *z*-dimension (Fig. 7c) to be the result of surface corrugation and offset Ga_2_ dimers, as seen in the phase-change structures (Fig. 1b and c).

For the penta-layer system, vague layering is seen in the *x*-dimension (Fig. 7d), however, no layering is seen in the *y*-dimension (Fig. 7e). Despite layering in the *x*-dimension, MSD_*x*_ is roughly equal to MSD_*y*_ (Fig. 6b). We expect that MSD_*x*_ is high because while layers are seen in the *x*-dimension they are transient (Fig. S6a†). Inter-planar layers are very clear (Fig. 7f) and correspond well to the low MSD_*z*_ (Fig. 6b).

Finally, for the hexa-layer system, no ordering is seen in the in-plane single coordinate atomic trajectories (Fig. 7g and h) which is reflected in approximately equal MSD_*x*_ and MSD_*y*_ (Fig. 6c). Interplanar layering is clear (Fig. 7i) and this is reflected in MSD_*z*_ (Fig. 6c).

Observing layers in the *z*-dimension corresponds to layering parallel to the surface; a well-known phenomenon in liquid metal surfaces.^36,56–59^ Our results show that parallel surface layering at the Ga/vacuum interface is present in the quad-, penta- and hexa-layer transitional 2D Ga structures and therefore this ordering does not appear to be thickness dependent.

**Fig. 8:**
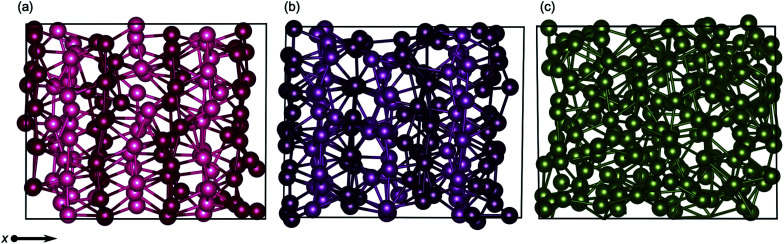
Snapshots of transitional structures for (a) quad-layer (450 K), (b) penta-layer (340 K) and (c) hexa-layer (440 K) structures. Atoms in successive *x*-layers are differentially shaded to guide the eye. No *x*-ordering is seen in the hexa-layer system. Black boxes show the unit cell.

Layering in the *x*-dimension means that the quad-, and for a brief moment in time, penta-layer, systems are ordering perpendicular to the Ga/vacuum interface. This phenomenon has, to date, only been deduced experimentally.^60,61^ Furthermore, the current study is, to the authors' knowledge, the first report of perpendicular layering at a liquid metal/vacuum interface. The appearance of perpendicular ordering at the Ga/vacuum interface at a minimum thickness of four and a maximum of five atomic layers illustrates that this structuring is highly sensitive to slab thickness (Fig. 8). An experimental study proposed that perpendicular layering at a Ga/C(111) interface could be attributed to the alignment of Ga_2_ dimers imposed by the C(111) surface.^60^ While we do not have an opposing crystal to dictate the surface structure nor covalently bound Ga_2_ dimers, we expect that the formation of a Ga surface at the Ga/vacuum interface imposes ordering on the layers beneath.

Finally, the ordering exhibited by these systems both parallel and perpendicular to the surface was examined in detail to ascertain if it is, in fact, the well known hexatic phase of 2D melting.^38–42^ We are, unfortunately, unable to conclusively diagnose the hexatic phase, due to the trade-off between simulating the “quasi-long range” effects required for a positive identification of the hexatic phase while still remaining computationally tractable. Despite this limitation, the orientational order correlation parameter, *g*_6_(*r*), was calculated up to 20 Å in the *xy* plane. There are limitations in defining an *xy* plane, particularly for the quad-layer structure where the planes are corrugated (Fig. 1b), therefore “in-plane” is defined as being within a *z* range of atom *i* to which the *g*_6_(*r*) parameter is measured. For the quad-layer system, hexatic-like ordering is observed through the melting transition (Fig. S8a†). In contrast to this, the solid and liquid penta-layer systems did not exhibit a high-degree of hexatic-like ordering, however, around the melting temperature (340 K) hexatic-like ordering appeared (Fig. S8b†). For the hexa-layer structure, hexatic-like ordering was observed in the solid but not through the melting transition nor in the liquid (Fig. S8c†). Importantly, we note that more definitive layering the *x*-dimension does not correspond to a higher degree of hexatic-like ordering. Therefore, *x*-ordering is not diagnostic of a hexatic-phase melting transition but rather is a structural feature of thin 2D Ga systems.

For all of these thin 2D Ga systems, parallel ordering inherent at finite thickness results in a reduction in entropy of the liquid. This reduction in mobility likely explains the raised *T*_melt_ of these systems, in the same way that is observed for quasi-2D liquid Ga nanoclusters.^29^ Emergence of perpendicular ordering in the quad- and penta-layer systems seen here will also have entropic effects, however this ordering is more equally distributed across both the solid and liquid phases. Therefore, the entropic effects of perpendicular layering are not reflected in the Δ*S* upon melting, thus explaining why the quad- and penta-layer systems can exhibit such different melting temperatures. However, the emergence of this perpendicular ordering for these two systems of atomically-precise thickness—four and five layers—is evidence of how sensitive structure is to thickness, which we see reflected in highly variable melting temperatures.

## Conclusions

The thermal stability of 2D Ga four, five and six atomic layers thick is considerably greater than the bulk thermal stability. Furthermore, the electronic structure of these systems is fully metallic for all, thus removing the necessity for the thickness of the 2D system to be atomically resolved when synthesising these materials for electronic applications. We propose that the change in thermal stability of the 2D structure can be estimated from the difference in stability due to thickness up to 10 atomic layers thick. For the first time, layering both parallel and perpendicular to a liquid metal/vacuum interface is reported. We determine that, despite ordering in the *x*-dimension, this is not indicative of a hexatic-phase melting transition. These results demonstrate both the dramatic variability of the stability of 2D Ga as a function of thickness and provide the prospect of synthetic techniques finding thicker forms of 2D Ga that are stable at higher temperatures.

## Conflicts of interest

There are no conflicts of interest to declare.

## Supplementary Material

NA-003-D0NA00737D-s001
