# Supplementary material for: Modulating the thermal and structural stability of gallenene via variation of atomistic thickness
Source: Nanoscale Adv. 2020 Dec 23;3(2):499–507. doi: 10.1039/d0na00737d (PMC9418766; doi:10.1039/d0na00737d)
Supplement: NA-003-D0NA00737D-s001 [file NA-003-D0NA00737D-s001.pdf]

Electronic Supplementary Material (ESI) for Nanoscale Advances.
This journal is © The Royal Society of Chemistry 2020

Supplementary Material

Modulating the thermal and structural stability of gallenene via variation of atomistic thickness

S. Lambie, K. Steenbergen, N. Gaston

November 12, 2020

Ground state optimisation

In order to determine the surface termination that should be used for seeding *ab initio* molecular dynamics (AIMD) simulations at finite temperature, four different quad-layer structures across both α and β phases of bulk Ga were optimised to find the lowest energy ground state structure. Three of the four structures were cut from the α-Ga phase and one was cut from the β-Ga phase. The three α-Ga structures are analogous to those found by Kochat *et al.* and labelling is kept consistent with their systems.[20] We choose these terminations as they have been found to be experimentally stable, in preference over all other surface terminations thus removing the requirement to test all surface terminations. That being said, we also trial one quad-layer β-phase termination in order to check that we are not missing a lower energy state.

Initial bulk crystals were taken from the Crystallography Open Database α-Ga with unit cell dimensions of 4.527 × 7.645 × 4.511 Å was used for α-Ga based systems[11] and β-Ga with unit cell dimensions of 2.791 × 7.890 × 3.286 Å was used for β-Ga based structures.[?]

Three of the four structures were cut from the α-Ga phase and one was cut from the β-Ga phase. The three α-Ga structures are analogous to those found by Kochat *et al.* and labelling is kept consistent with their systems.[20] Each surface is periodic in x and y dimensions, and separated from the next unit in the z direction by 30 Å of vacuum. All surfaces were four atomic layers thick and all unit cells contained 8 atoms. Below details a list of the terminations of the four quad-layer structures. Graphic representations of initial and optimised structures can be found in Table S1.

- *Surface 1:* α-a_{100}: Cut along the (100) plane of bulk α-Ga
- *Surface 2:* α-$b_{010}a$: Cut along the (010) plane of bulk α-Ga, cutting through covalent dimers to generate the surface. This results in a metallic layer, followed by a Ga_2 dimer, followed by a metallic layer.
- *Surface 3:* α-$b_{010}b$: Cut along the (010) plane of bulk α-Ga, cutting through metallic planes to generate the surface. This results in two layers of stacked Ga_2 dimers.
- *Surface 4:* β-a_{001}: Cut along the (001) plane of bulk β-Ga

Table S1: Relative electronic stability per atom and electron localisation function (ELF) of four trialled ground state structures of quad-layer Ga. Isosurface level is set to 0.80 and bonds less than 2.7 Å are shown.

System	Rel. Energy [eV atom^{-1}]	Initial	Optimised	ELF
α-a$_{100}$	0.032	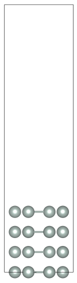	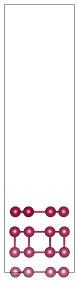	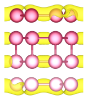
α-b$_{010}$-a	0.000	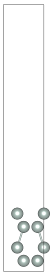	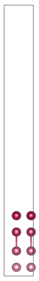	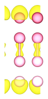
α-b$_{010}$-b	0.006	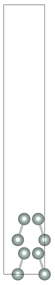	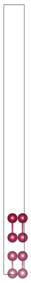	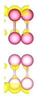
β	0.085	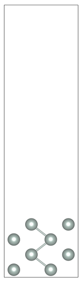	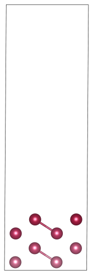	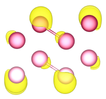

In all of the α-Ga surfaces, the Ga_2 dimers align with the z dimension upon optimisation. The only structure to display covalency, from the ELF, upon optimisation is α-b$_{010}$a. The α-b$_{010}$a is the lowest energy ground state structure after optimisation. However, the relative energies of all optimised ground-state structures are within 0.1 eV atom^{-1} making them competitive structures (Table S1).

Profile of average energy as a function of average temperature during annealing

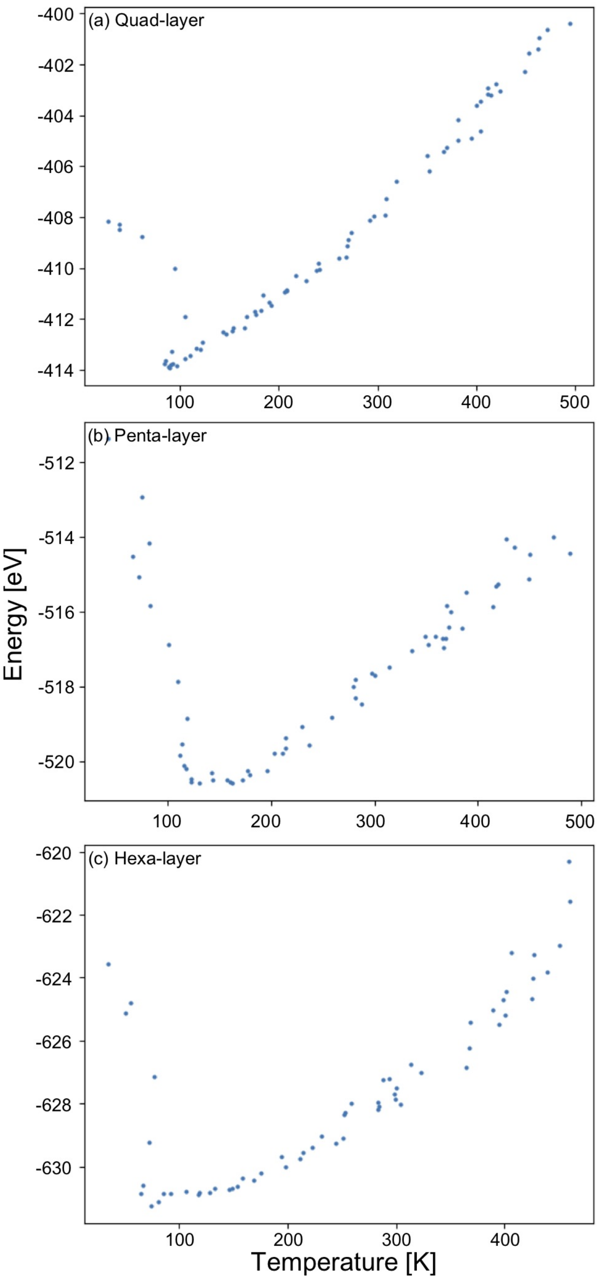

Figure S1: Average energy as a function of average temperature for *(a)* quad-layer, *(b)* penta-layer and *(c)* hexa-layer over the course of the annealing simulations.

Parameters defining ground state structures

Table S2: Parameters of bulk α-Ga and the corresponding parameters for lowest energy structures for quad-layer, penta-layer and hexa-layer.

System	Dimer distance [Å]	Dimer angle [°]	Buckled plane height [Å]
Bulk α-Ga	2.44	16.9	1.49
Quad-layer	2.68	1.03	1.86 (min); 3.39 (max)
Penta-layer	2.65	0.69	2.41
Hexa-layer	-	-	-

Radial Distribution Function of lowest energy structures

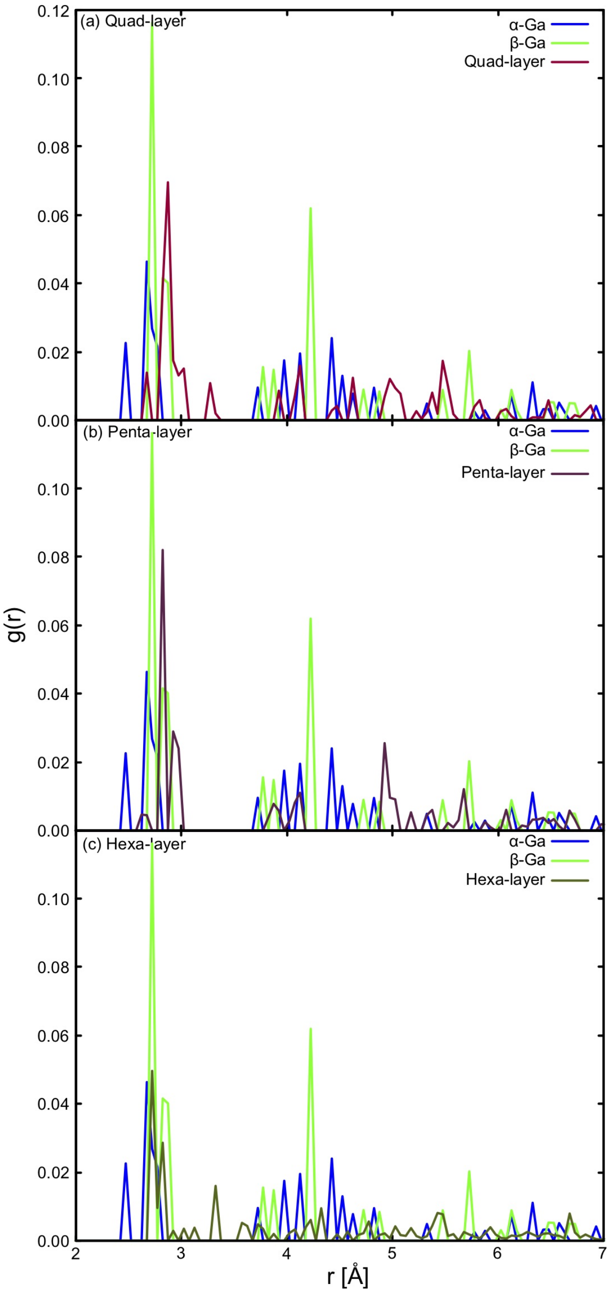

Figure S2: Radial distribution function of the optimised lowest energy structures of *(a)* quad-layer, *(b)* penta-layer and *(c)* hexa-layer compared to bulk α- and bulk β-Ga.

Electron Localisation Function of lowest energy structures

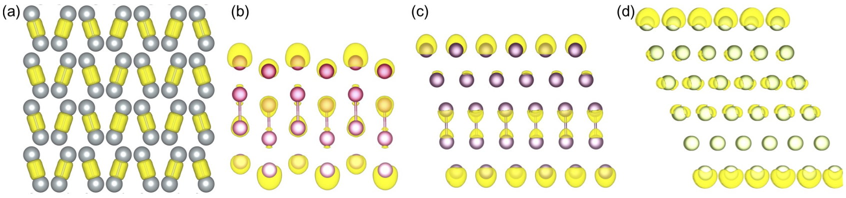

Figure S3: Electron localisation functions for optimised lowest energy structures of *(a)* bulk α-Ga and resulting from the phase change observed during annealing of *(b)* quad-layer, *(c)* the penta-layer and *(d)* the hexa-layer structure. Isosurface level set to 0.80 and the bond length cut off was set to 2.7 Å.

Band structure of lowest energy structures

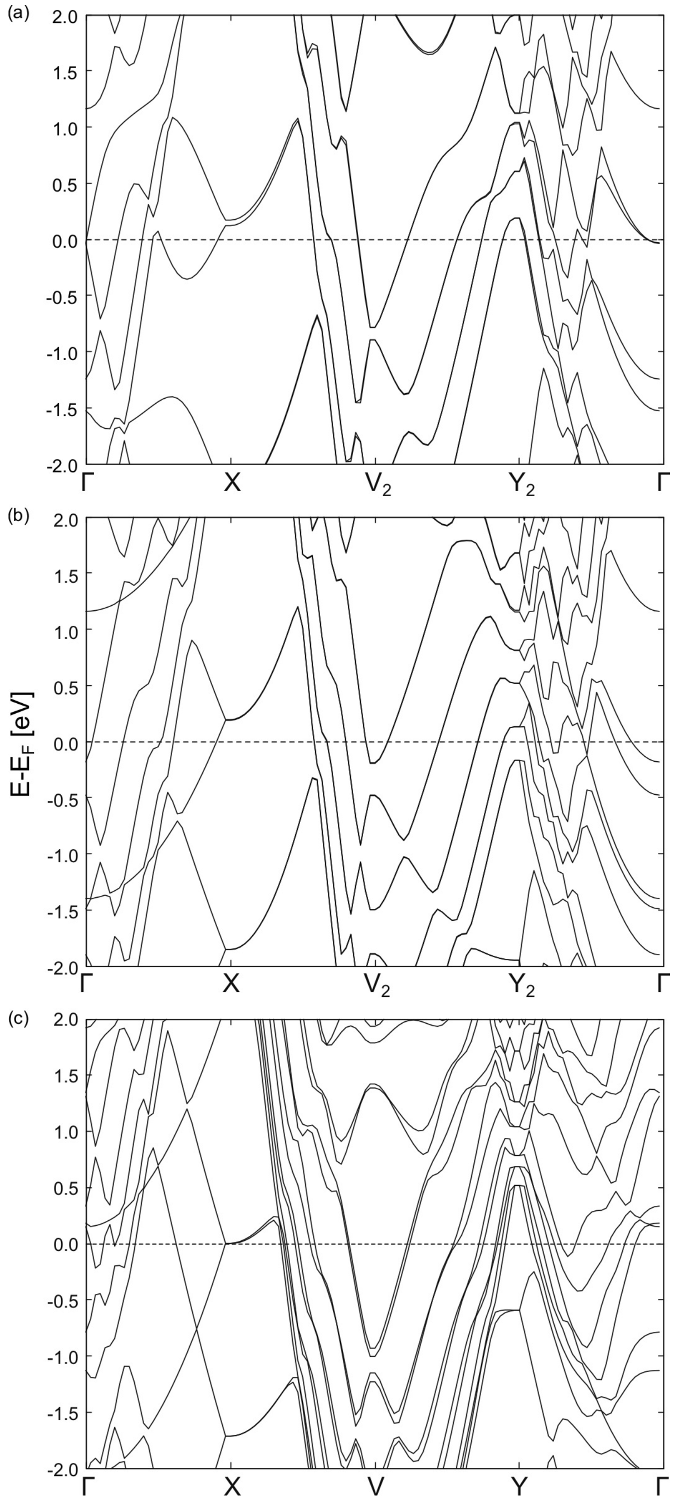

Figure S4: Band structure of optimised lowest energy *(a)* quad-layer *(b)* penta-layer and *(c)* hexa-layer structures.

Electron Localisation Function analysis as a result of increasing the number of layers in the 2D system.

Table S3: Increasing the number of layers to examine the convergence to the bulk alpha-Ga structure using electron localisation function. Bonds less than 2.6 Å are shown. Isosurface value is set to 0.77. Red atoms denote the quad-layer system, purple represents penta-layer, green represents hexa-layer and blue atoms denote all other optimised systems.

Bulk	2 layers	3 layers	4 layers
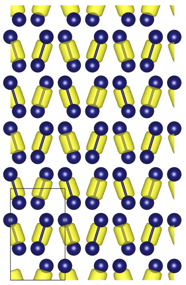	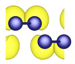	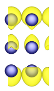	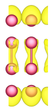
**5 layers**	**6 layers**	**7 layers**	**8 layers**
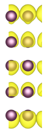	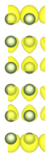	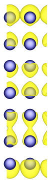	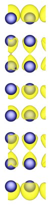
**9 layers**	**10 layers**	**11 layers**	**12 layers**
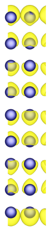	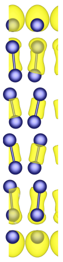	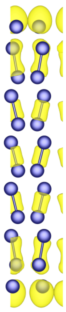	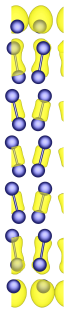

Single coordinate atomic trajectories

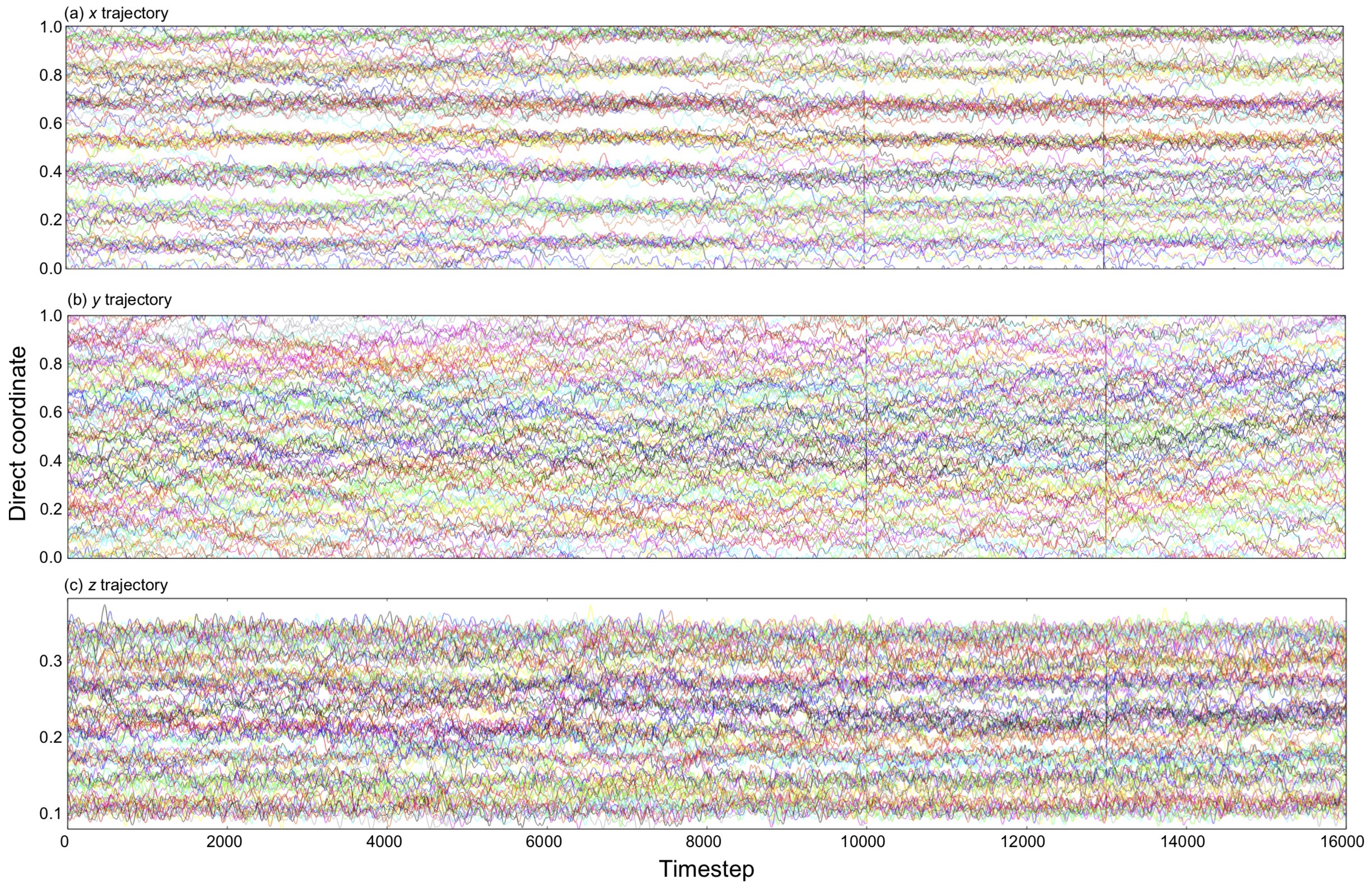

Figure S5: x, y and z coordinates as a function of timestep for all atoms in the quad-layer simulation at a thermostatted 450 K (T_{melt}: 457 K) molecular dynamics run, using the nVT ensemble. Layers are clearly seen in the (a) x dimension, not seen in the (b) y dimension and are apparent in the (c) z dimension.

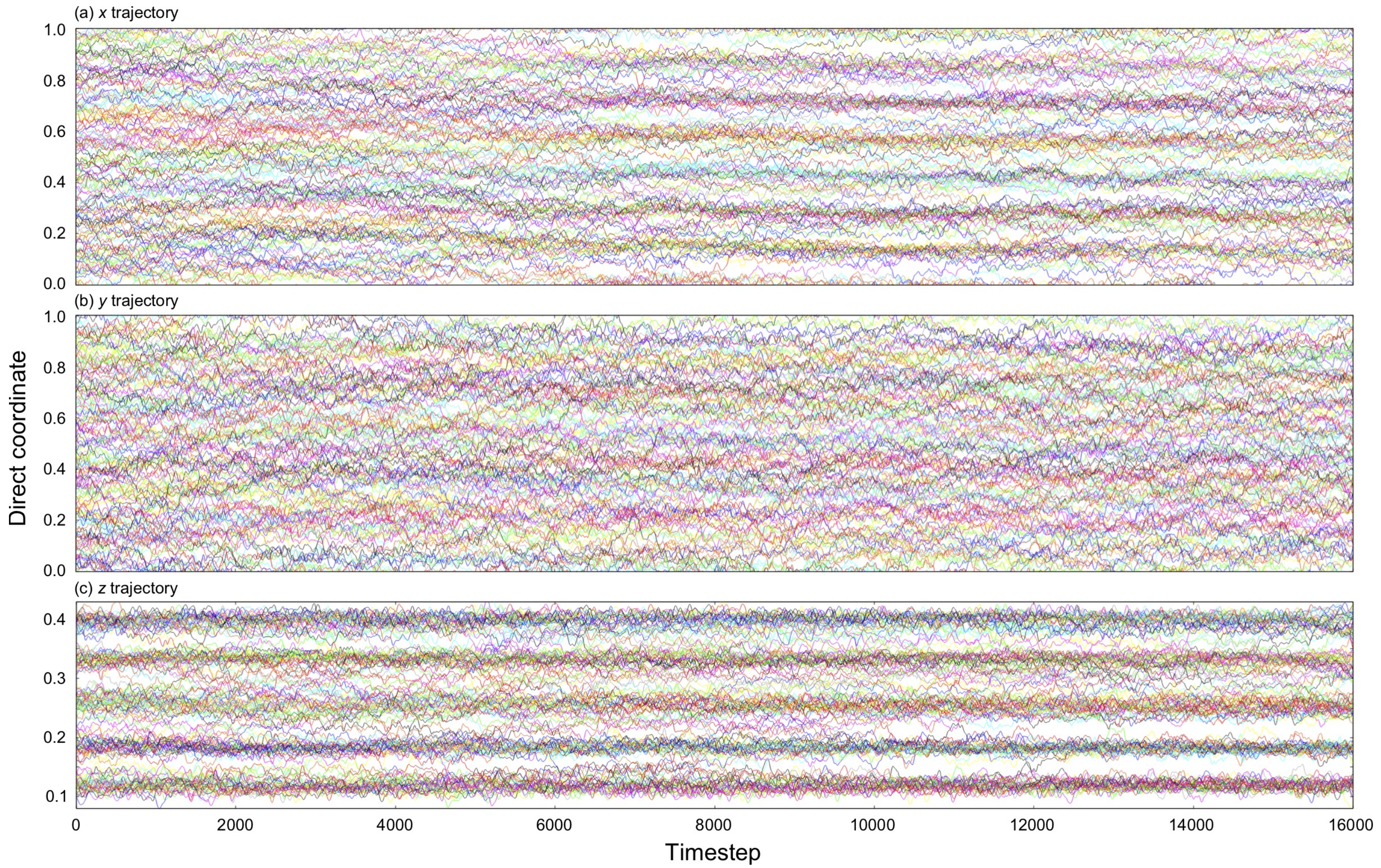

Figure S6: x, y and z coordinates as a function of timestep for all atoms in the penta-layer simulation at a thermostatted 340 K (T_{melt}: 350 K) molecular dynamics run, using the nVT ensemble. (a) Some layering in the x dimension can be seen, (b) no distinct layers are seen in the y dimension but (c) are apparent in the z dimension.

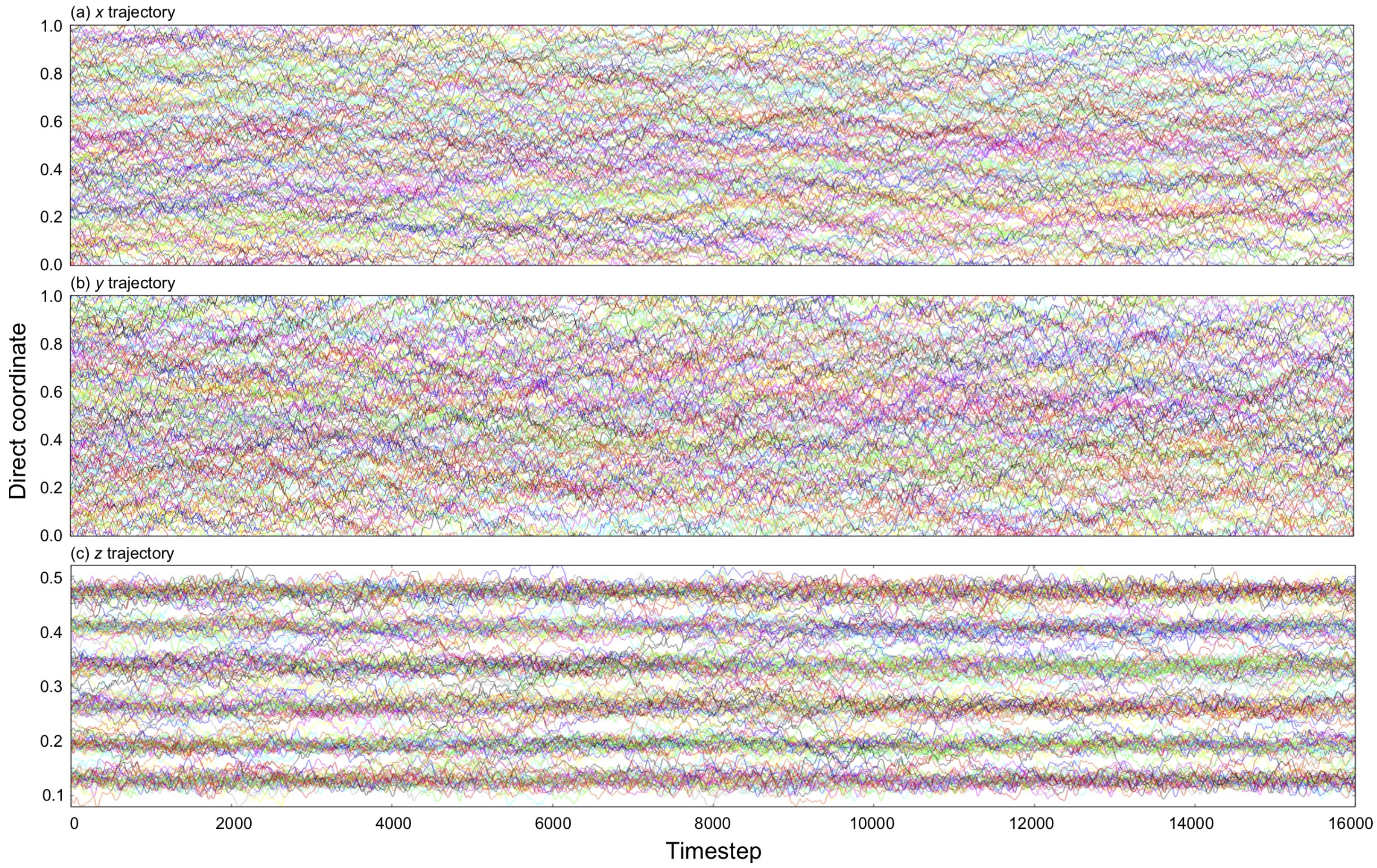

Figure S7: x, y and z coordinates as a function of timestep for all atoms in the hexa-layer simulation at a thermostatted 440 K (T_{melt}: 433 K) molecular dynamics run, using the nVT ensemble. No layers are seen in the (a) textitx or (b) y dimensions but are apparent in the (c) z dimensions.

Hexatic-ordering calculations

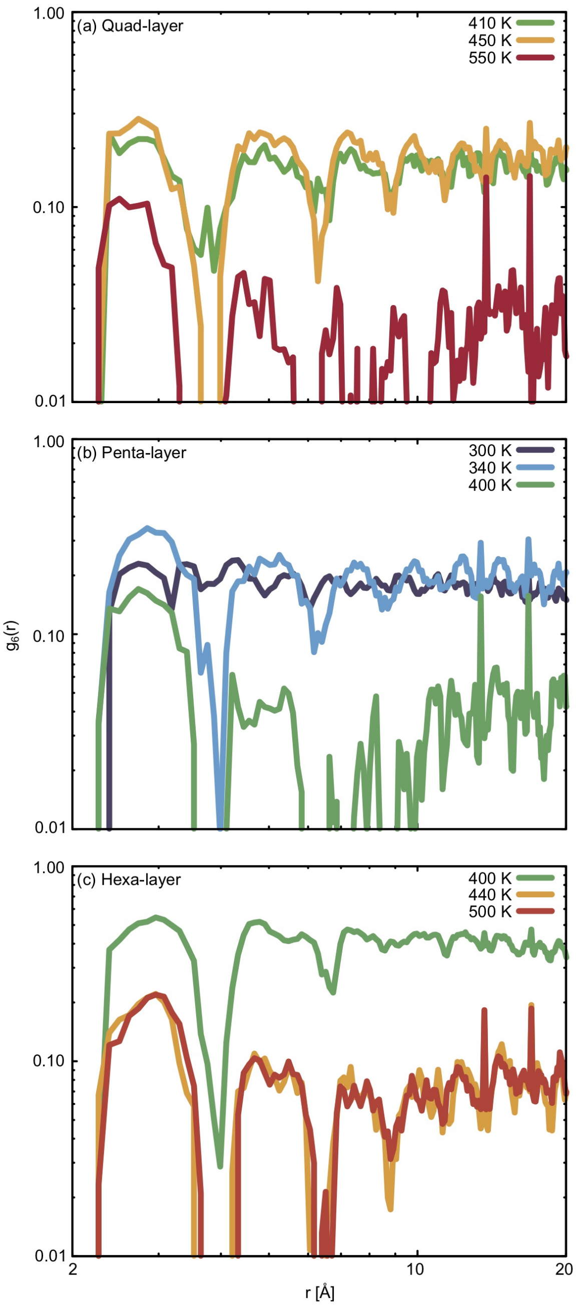

Figure S8: Orientational order correlation function ($g_6(r)$) as a function of temperature for *(a)* quad-layer, *(b)* penta-layer and *(c)* hexa-layer. We use the lowest finite temperature simulation as the high temperature "solid" phase and the highest temperature finite temperature simulation as the "liquid" phase. The transitional structure between the solid and liquid comes from the finite temperature in closest proximity to the melting temperature; 450 K for the quad-layer system (T_{melt}: 457 K), 340 K for the penta-layer system (T_{melt}: 350 K) and 440 K for the hexa-layer system (T_{melt}: 430 K).

References

[20] Kochat, V., Samanta, A., Zhang, Y., Bhowmick, S., Manimunda, P., Asif, S., Stender, A., Vajtai, R., Singh, A., Tiwary, C., Ajayan, P., Atomically thin gallium layers from solid-melt exfoliation. *Sci. Adv.* **2018**, *4*, 1701373.

[11] Bradley, A., The cystal structure of gallium. *Z. Kristallogr.* **1935**, *91*, 302–316.

[] Bosio, P., Defrain, A., Structure cristalline du gallium-β. *Acta. Cryst., Sect. B: Struct. Crystallogr. Cryst. Chem.* **1969**, *25*, 995.
